# To end disease tomorrow, begin with trials today: Digital strategies for increased awareness of a clinical trials finder

**DOI:** 10.1017/cts.2019.404

**Published:** 2019-08-20

**Authors:** Rebecca N. Jerome, Leah Dunkel, Nan Kennedy, Erik J. Olson, Jill M. Pulley, Gordon Bernard, Consuelo H. Wilkins, Paul A. Harris

**Affiliations:** 1 Vanderbilt Institute for Clinical and Translational Research, Vanderbilt University Medical Center, Nashville, TN, USA; 2 Department of Medicine, Vanderbilt University Medical Center, Nashville, TN, USA; 3 Department of Internal Medicine, Meharry Medical College, Nashville, TN, USA

**Keywords:** Clinical trials, patient information needs, social media, public service announcements, recruitment

## Abstract

**Introduction:**

Individuals experiencing different medical conditions, as well as healthy volunteers, may often be interested in trial participation, and researchers similarly need to find participants to advance medical knowledge. The ResearchMatch (RM) Trials Today clinical trial searching tool leverages clinicaltrials.gov data to enable potential participants to look for trial opportunities relevant to their situation. To facilitate expanded use of this tool, we undertook a national digital public awareness campaign to increase awareness of Trials Today among members of the general public.

**Methods:**

The awareness campaign promoted Trials Today using Facebook and digital banner messages in 2017, encompassing nine cities across the USA. The digital strategy was complemented by print media in several outlets. We employed descriptive statistics to summarize campaign metrics and site usage data during the campaign.

**Results:**

The campaign was successful in increasing visits to Trials Today, with 142,303 sessions logged during its run, as compared to pre-campaign data indicating 104,688 total sessions during the entire 2-year period since the site’s inception. The city-specific click-through rate for all digital impressions, combining Facebook and banner messaging, ranged from 0.50% to 1.09%, resulting in a cost-per-click range of $0.69–$1.15. In addition, visitors conducted 29,697 searches and viewed individual trial records 173,512 times.

**Conclusion:**

The public awareness campaign was successful in increasing use of the RM Trials Today clinical trial searching tool. Our findings support the value of digital media messaging as a cost-effective vehicle for promoting clinical trial awareness, especially for chronic ailments.

## Introduction

Advances in maintaining and restoring human health, including new and more effective strategies for preventing and treating various conditions, require the design and conduct of productive clinical research. These studies rely on significant contributions from researchers and participants, among other stakeholders. A successful clinical trial cannot be completed without connections between members of the general public who may want to participate in research and the researchers who are looking for participants just like them. Publicly available resources such as clinicaltrials.gov provide information about existing trials. However, such sites are designed primarily to promote research transparency [1–6], rather than as tools to encourage connections between potential participants and research studies of interest. Moreover, the clinicaltrials.gov site has many components and serves multiple functions, so may present challenges to lay individuals who are merely seeking information and opportunities related to currently enrolling trials.

Connecting potential participants with studies for which they may be eligible has long been a key mission of our ResearchMatch (RM) community [7], a national volunteer-to-researcher matching platform. To further address the general public’s need to more easily and proactively locate clinical studies of interest, our team at the Vanderbilt Institute of Clinical and Translational Research launched an online tool in 2015, within the RM platform, called Trials Today [8]. Trials Today uses a lay-friendly, question-driven search interface to list currently recruiting trials derived from data downloaded daily from clinicaltrials.gov [9]. RM and Trials Today are supported in part by the National Institutes of Health (NIH).

Trials Today enables potential participants to search for clinical trials themselves and initiate contact with researchers. While RM leverages established national networks and other relationships to connect with researchers, reaching the public at large to publicize availability of the new tool required an entirely different investment of effort and financial resources. Indeed, funding to support media outreach on a large scale is not readily available within traditional federal funding programs. While evidence shows that many people desire to participate in trials [10,11], lack of knowledge about available trials contributes to persistent and pervasive recruitment challenges [12,13].

Public service announcement (PSA) strategies have an established history in the USA for promoting public service messages within the national and regional media (e.g., print, television, radio, internet). While PSAs began as unpaid advertisements from a government agency or non-profit organization promoting a message, activity, or program [14,15], the proliferation of PSAs, competition for broadcast placement, and the increasing prominence of digital media have impelled many organizations to pay for placement to ensure their message reaches a sizeable number of people [16]. With the goal of fostering public awareness of the Trials Today platform as a no-cost service to potential research participants, we designed, produced, and implemented a PSA campaign.

## Methods

### Branding and Marketing Strategy

The existing RM volunteer base was surveyed regarding potential names and the ideal relationship between the tool and RM, indicating a preference for the name “Trials Today.” Graphic continuity between RM and Trials Today design concepts was created, including similar visual style and complementary color palettes. As a part of this campaign, we worked alongside the marketing firm Red Deluxe, who provided guidance on strategy, developed creative elements of the campaign, and assisted with purchase and delivery of PSAs.

### Aligning with the Public Good

Our goal of promoting awareness of clinical trials and the existence of Trials Today aligns tightly with the broader mission of RM to facilitate connections between researchers and participants to support research that contributes to advances in health and healthcare. PSA concepts for both web distribution and those slated to run in local print media were designed to avoid the appearance of an industry-sponsored clinical trial and be straightforward and understandable across audiences. Additionally, messages used in the Facebook component of the campaign were developed in adherence to Facebook’s advertising guidelines.

### PSA Concept Development

We developed general graphics focused on Trials Today plus condition-focused concepts to most effectively connect with potential users of the trial searching tool. Concepts employed altruism-based messages (e.g., “To end Diabetes tomorrow, start with Trials Today”) with the goal of encouraging individuals with certain chronic conditions, as well as healthy volunteers, to contribute to the health of future generations by participating in clinical trials. We first chose specific conditions based on frequently searched topics in Trials Today, such as heart disease, Alzheimer’s disease, and lupus. To leverage synergy with other NIH initiatives, we also incorporated conditions that are the focus of clinical trials within the Trial Innovation Network [17], a collaborative initiative addressing inefficiencies within clinical trials, and the CTSA Consortium [18], a national network of institutions focused on improving translational research.

**Fig. 1.**
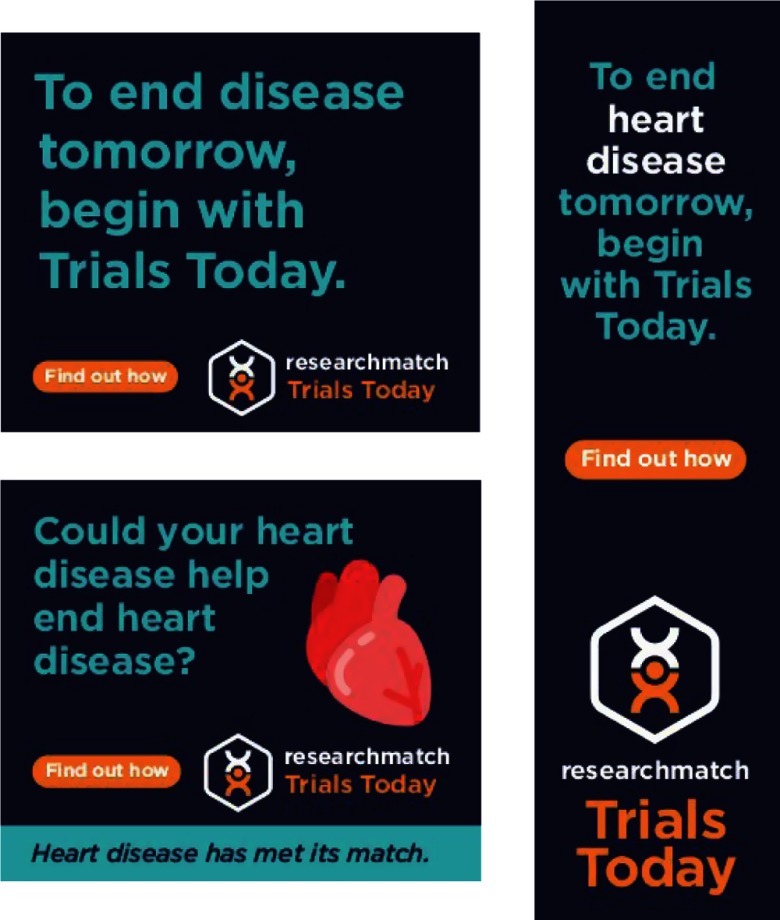
Sample newsfeed and banner graphics used in digital awareness campaign.

We developed a selection of banner and newsfeed graphics for use in eventual promotion in the digital phase of our campaign and print complements (see Fig. 1 for example). In addition, we developed a postcard mailer and 30-second animated video designed for digital distribution through social media.

### Selection of Geographic Areas for the Campaign

**Fig. 2.**
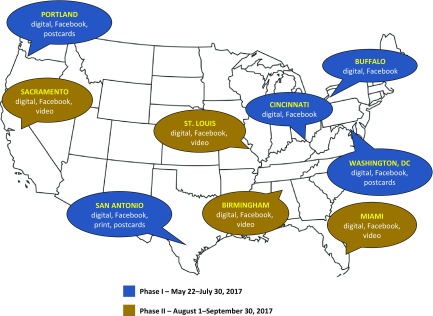
Media campaigns by market.

The team selected target cities based on geographic and population diversity, including areas with large subgroups from traditionally underrepresented populations (see Fig. 2 for divisions by phase). Preference was shown to locations with an existing CTSA site, Trial Innovation Network study location, or a registered RM partner institution. Using this strategy, the team sought to ensure synergy between Trials Today, the CTSA Consortium, and greater RM community. For disease-specific messages, areas were chosen based on certain criteria including: (1) For banner and Facebook advertisements, the existence of actively recruiting studies for the disease in the geographic area and (2) For Facebook, the disease was required to meet Facebook reach threshold requirements (e.g., frequency of users in the geographic area). In the case of rare conditions such as pulmonary sarcoidosis, Facebook targeting was not feasible given the small available population.

### Campaign Promotion

The campaign included a combination of digital and Facebook PSAs, complemented with print PSAs, direct mail, and content uploaded to a PSA clearinghouse which eventually resulted in inclusion in several magazines, including *Time* (August 28, 2017), *OK!* (August 14, 2017), *Money* (November 2017), and the digital edition of *Redbook* (September 2017). We targeted the delivery of our PSAs to specific Facebook users based on those users’ self-identified geographic location, demographic details (e.g., sex, race, age), page likes, and searches. This part of our campaign strategy focused on Facebook only; we did not elect to include Instagram, to allow assessment of Facebook as a standalone channel for awareness efforts. Users with relevant previous search history (e.g., heart disease) saw digital banners displayed on websites such as WebMD. The purpose of targeting by demographic segments was to foster inclusion of underrepresented groups.

During the campaign, we also employed a tracking pixel (navigation tracker), placed on the Trials Today landing page, which allowed us to gather information about referring websites and de-identified general demographics profiles of visitors, as well as data regarding visitor paths through the website (e.g., searching, viewing results).

### Ethical Considerations

While framing this PSA plan, we considered at length the potential privacy and confidentiality issues, as well as estimation of risk and benefit. Because clinical trial recruitment via online campaigns is relatively new, ethical guidance for researchers is limited [19–21].

One ethical consideration in conducting this study was whether, by placing a Facebook post or web banner making mention of a condition or disease, there might be an unintended audit log established that could be exploited to breach confidentiality. When a user clicks on an ad referencing a specific disease, a data trail may be unavoidably initiated and preserved.

In this case, however, our targeted group had already expressed an interest in or connection to the specific disease in the past, through previous internet and/or Facebook activity (e.g., searching for information on a condition or a related treatment or following Facebook pages related to a condition or its treatments). It was this previous online history, in fact, that enabled us to focus our campaign on such individuals in the first place. These individuals had, in a sense, already “disclosed” this health-related information online in a way that was tracked. As a protection against this concern, Facebook rejects the wording of paid promotions that appear to target specific characteristics of individuals, including that they suffer from a particular disease [22].

An additional ethical consideration was our placement of a tracking pixel within the Trials Today site during the campaign. Such pixels acquire information about IP addresses and known demographics such as age, sex, and general location. Some data protection advocates are wary of pixels because they collect such information without express permission of the user [23]. Importantly, however, the information collected is shown only in aggregate form for demographic segments that contain a minimum of 100 adults [24]. Further, we placed this pixel only on our main Trials Today landing page; it was not included deeper within the site, such as the mediated search page on which an individual enters further search detail such as age, condition, or other preferences.

We also considered risk-benefit in our approach. The risk of a breach of personal, sensitive data collected as a result of clicking on our awareness messages was small, all online digital activity carries some level of privacy risk. Disclosure of such information could result in harms relating to reputation, employment, or insurance due to discrimination or stigmatization [25]. Conversely, under-enrollment in clinical trials results in the loss of valuable knowledge to improve health care, and furthermore exposes individuals participating in unfinished trials to unnecessary risks [26].

While it is not the purpose of this paper to delve deeply into privacy concerns inherent in advertising on the Internet, our team did examine and discuss these issues in advance. Bender *et al.* argue that any measures to protect privacy should be commensurate with the level of data sensitivity [25]. Hibbin *et al.* advocate for a flexible, nuanced assessment of social media data use and consent [27]. We ultimately weighed the potential privacy intrusions of our outreach against the value of users learning about clinical trials that could potentially provide a health benefit to them or others. As a final step before enacting this plan, we sought and received approval from the Vanderbilt Institutional Review Board (IRB #090207).

### Campaign Implementation

Phase 1 of the campaign ran from May 22 to June 30th, 2017 and included Buffalo, New York, Cincinnati, Ohio, Washington DC, San Antonio, Texas, and Portland, Oregon. This phase focused on disease-specific messages around chronic diseases such as diabetes, kidney disease, heart disease, Crohn’s, Alzheimer’s, COPD, asthma, and pulmonary sarcoidosis. Our Trials Today print PSA also ran in the *San Antonio Express-News* (June 18, 2017; circulation 230,000) and *San Antonio* magazine (July and August 2017 issues; circulation 29,500). To leverage visibility in other popular media, our awareness materials were also added to a national PSA download center used by magazines and made outreach calls to PSA directors for major magazine chains. In addition, we distributed postcard mailers to 15,000 residents in San Antonio, 10,000 in Portland, and 5,000 in Washington DC.

Phase 2 ran from August 1 to September 30, 2017 and included Miami, Florida, Birmingham, Alabama, St. Louis, Missouri, and Sacramento, California. In this phase, general concepts ran alongside disease-specific graphics for diabetes, heart disease, Alzheimer’s, and lupus. This phase also included postcard mailers and the 30-second animated video.

### Role of Community Feedback

In addition to a collaboration with Red Deluxe, the Vanderbilt team sought ongoing feedback from community members throughout development and implementation of the campaign. The STAR Clinical Research Network [28] reviewed many of the proposed graphics and recommended changes to language and greater diversity in imagery (e.g., using varying skin colors in graphics including hands). During Phase 2 of the campaign, the Recruitment Innovation Center Community Advisory Board provided input on a 30-second animated video, recommending slowing the voice-over and including a more diverse representation of clinical research (e.g., not limiting to drug trials). The team implemented recommendations to the video following the end of Phase 2.

### User Survey

To obtain feedback on usability of the site from the influx of new visitors, we included a feedback survey during Phase 2 of the campaign, which appeared as a pop-up on the next page load after a visitor was on the site for a minute and on 10% of the pages that were loaded after a minute among visitors who viewed multiple pages on the Trials Today site. Trials Today user satisfaction data were collected and managed using the REDCap electronic data capture tool hosted at Vanderbilt University Medical Center [29]. Users provided feedback on ease of use, purpose of the search, likelihood of reaching out to a study contact, along with basic demographic information.

### Descriptive Analytics

The team monitored usage of the tool and reach of PSAs throughout the campaign using data provided by Red Deluxe as well as Trials Today server statistics captured using Google Analytics.

## Results

**Fig. 3.**
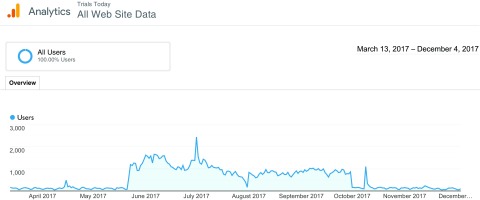
Number of Trials Today users during the two phases and pre-and post-campaign.

During the two-year period from the inception of Trials Today in March 2015 through the day before the launch of the PSA campaign in May 2017, Trials Today logged a total of 104,688 sessions. During the campaign, the site experienced a notable increase in traffic. Over the course of our two-phase public awareness campaign, the Trials Today site logged 142,303 sessions, with 87,794 in Phase 1 and 54,322 in Phase 2. Fig. 3 charts the number of users over the course of the two messaging campaigns, as well as pre- and post-campaign. Usage increased dramatically during message promotion, with further spikes occurring when a RM newsletter, containing a link to the Trials Today site, was emailed to subscribers during July and October 2017.

### Digital and Print Promotion Campaign Metrics

The click-through rate from all digital impressions, combining Facebook and digital banner promotions, ranged from a low of 0.50% in Washington DC to a high of 1.09% in San Antonio, resulting in a cost-per-click of $0.69 in San Antonio (the lowest) to $1.15 (the highest) in Sacramento (Table 1). The video messaging was particularly effective in terms of reach. The average view-through rate in markets showing the video was nearly 40%, meaning that almost two in five of those exposed to the video watched it through to the end. Moreover, click-through rates to the Trials Today site of 0.60% or better were recorded in four of the five video markets (Table 2).

**Table 1.** Digital awareness messaging metrics by phase and city

	Phase I 5/22/17–7/30/17				Phase II 8/1/17–9/30/17				
	San Antonio, TX	Buffalo, NY	Portland, OR	Cincinnati, OH	Washington, DC	Sacramento, CA	Birmingham, AL	Miami, FL	St. Louis, MO
**Impressions**^a^	4,617,101	3,073,617	3,530,063	4,283,995	2,473,126	1,980,684	1,925,691	2,675,519	2,469,008
**Clicks**^b^	50,316	16,551	19,408	27,077	12,340	12,389	13,764	27,771	16,361
**CPM**^c^	$7.47	$5.37	$5.66	$6.13	$4.95	$7.21	$7.14	$8.69	$7.58
**CTR** ^d^	1.09%	0.54%	0.55%	0.63%	0.50%	0.63%	0.71%	1.04%	0.66%
**CPC** ^e^	$0.69	$1.00	$1.03	$0.97	$0.99	$1.15	$1.00	$0.84	$1.14

a Total message impressions.

b Total number of clicks.

c Cost per thousand advertising impressions.

d Click-through rate (percentage of total impressions in which message was clicked on).

e Cost per click.

**Table 2.** Video metrics by market

	Sacramento, CA	Birmingham, AL	Miami, FL	St. Louis, MO	Total
**Impressions**	144,646	139,149	144,971	139,397	568,163
**:03 Views**	111,181	106,351	110,323	105,081	432,936
**100% Views**	56,932	48,750	57,852	50,480	214,014
**VTR** ^a^	39.36%	35.03%	39.91%	36.21%	37.67%
**Clicks**	388	920	1,287	838	3,433
**CTR** ^b^	0.27%	0.66%	0.89%	0.60%	0.60%

a View-through rate (percentage of total impressions in which the full video was watched).

b Click-through rate (percentage of total impressions in which message was clicked on).

Click-through rates for disease-specific banner messaging did not vary dramatically across markets or disease topics—nearly all averaged less than 0.30% (Supplemental Table 1). Only COPD messaging in San Antonio (0.33%) and Alzheimer’s disease in Portland (0.34%) obtained slightly higher rates. The poorest click-through rates were found in St. Louis, where the response was especially low for heart disease (0.14%) and general concepts (0.16%). Facebook posts performed significantly better (Supplemental Table 2). Click-through rates ranged from lows of 0.66–0.79% for lupus in the three markets where it was tested, to highs of 3.19% for COPD in San Antonio and diabetes in Washington, DC. Overall, COPD and diabetes were the medical concepts that produced the highest response rates, while lupus generated the least amount of interest.

The awareness concepts we uploaded to the PSA clearinghouse were picked up by a number of print and digital publications of national reach, reaching a total audience of over 5 million and representing an approximate 3300% return on our investment in the print PSA clearinghouse, although there was no way to track the volume of accesses directly inspired by those materials. In San Antonio, the only city in which we purchased local print media placements, we reached approximately 289,000 people with an estimated cost per thousand impressions of $35.57.

### Search Activity

Trials Today Visitors conducted a total of 29,697 searches during the campaign, including 14,329 in Phase 1 (May–July 2017) and 15,368 in Phase 2 (August–September 2017). In addition, the site registered 83,819 views of specific clinical trials in Phase 1, and another 89,693 in Phase 2.

Table 3 describes user-reported reasons for using the Trials Today search tool, with those recently diagnosed with a disease/condition looking for treatment options (*n* = 7758, 26%) and those interested in being a healthy volunteer (*n* = 6777, 23%) predominating among visitors, as well as those seeking to use the advanced search option (*n* = 7521, 25%). The proportions of users selecting the various search options were similar between Phase 1 and Phase 2, though the advanced search option was two times more popular during Phase 2 as compared with Phase 1 (33% vs. 17%), suggesting that the proportion of more search-savvy users was higher during the second phase of our campaign.

**Table 3.** Trials Today search purposes, as reported in mediated search interface

User-reported reason for search	Phase 1 *n* (%)	Phase 2 *n* (%)	Total *n* (%)
I was recently diagnosed with a disease or condition and I am looking for treatment options, even those that are not yet approved	4052 (28)	3706 (24)	7758 (26)
I understand how clinical trials work and would like to fine-tune the search myself	2451 (17)	5070 (33)	7521 (25)
I am not looking for any treatments but I am interested in being a healthy volunteer for research studies that could help other people in the future	3745 (26)	3032 (20)	6777 (23)
I have/had a disease or condition and I am interested in finding studies that test new treatments	1920 (14)	1537 (10)	3457 (11)
I have/had a disease or condition and I have already tried the currently approved treatment options and they are not working/are no longer working for me	1307 (9)	1199 (8)	2506 (8)
I was recently diagnosed with a disease or condition and there are no available treatments at all	762 (5)	721 (4)	1483 (5)
I am not looking for any treatments but I am interested in studies that figure out ways to diagnose diseases, develop preventative measures, or develop screening processes	92 (1)	103 (1)	195 (1)
**Total (** ***n*** **)**	14,329	15,368	29,697

Note: “*n*” represents the number of searches in each category.

Table 4 illustrates the most popular searches during each phase of the campaign. Of note, there was 80% overlap between the top 10 most-searched topics between the two phases, with eight conditions appearing in both phases.

**Table 4.** Top 10 most frequently searched conditions

	Phase 1		Phase 2	
Condition	*n*	Rank within top 10	*n*	Rank within top 10
Alcohol	682	1	662	1
Multiple sclerosis	354	2	303	4
Arthritis	NA	NA	342	2
Lung cancer	266	3	NA	NA
Cancer	NA	NA	322	3
Depression	260	4	189	6
Methamphetamine dependence (disorder)	221	5	239	5
Diffuse large B-cell lymphoma	158	6	120	9
Anxiety	110	7	NA	NA
Healthy	110	8	137	7
Obesity	101	9	94	10
Idiopathic pulmonary fibrosis	71	10	135	8

Note: We combined overlapping concepts, including “Alcohol Addiction” and “Alcohol” to “Alcohol”, “Depression” and “Major Depression” to “Depression,1” and “Healthy” and “Healthy Adult” to “Healthy.” “*n*” represents the number of searches on each topic.

We also monitored the rate at which new volunteers signed up for membership in the RM community during the campaign; we observed no noticeable increase in new volunteer registrations during the Trials Today awareness campaign.

### User Satisfaction

Results of our Trials Today user satisfaction survey are detailed in the online supplement (Supplemental Figures 1–4). In brief, two-thirds (66%) of surveyed users agreed or agreed strongly that the tool was helpful, and 83% that it was easy to use. The large majority (94%) said they were seeking a clinical trial for themselves, and 62% reported being likely or very likely to contact one of the studies they found on the site. User comments regarding the site were largely favorable, although several suggestions for improvement were offered.

## Discussion

Access to clinical trials can be considered an important service in the public good. The messages we developed were intended to function as PSAs for those interested in pursuing participation in clinical research. Our campaign also experienced significant expansion beyond our initial investment. While this campaign included allocation of financial resources to the design and placement of PSA materials via social media and other digital channels, we also benefited from an exponential increase in the reach of the campaign when our messages were picked up for inclusion in a number of popular media outlets (e.g., *Time* magazine) at no added expense beyond our initial commitment.

While selection bias should always be a consideration when planning awareness campaigns, a notable upsurge in digital media usage across demographic groups over the past decade [30] provided confidence that social media could be used to connect with diverse populations. Facebook is the social media outlet with the largest number of active users worldwide, by a large margin [31]. Facebook-based efforts to recruit participants for online surveys and in-person clinical trials or interventions have been largely successful, especially in recent years [32–39], and particularly when compared to traditional print methods of recruiting such as newspaper ads, leaflets, posters, and mail [40–44]. Facebook reach can be especially effective for a geographically dispersed population [45,46]. Some studies have also been successful in recruiting participants through the use of internet messaging such as banner ads that are programmed by Google AdWords to appear based on user search terms [41,47–51].

Over the 19 weeks of our campaign to foster awareness of our Trials Today clinical trial searching tool, we leveraged visibility on multiple channels including Facebook, other internet PSA placements, and print media. We saw a dramatic increase in the number of visitors to the site, with a small but notable proportion employing the search feature to explore currently recruiting trials of potential interest to them. The data regarding the most-searched-for topics reflected a broad array of medical situations for which there is clear interest among potential participants, ranging from alcohol and substance abuse and depression to pulmonary conditions to other chronic diseases such as arthritis and obesity. This wide range of searched goals and topics further emphasizes the myriad purposes that may prompt consideration of trial participation among members of the general public.

Research investigators are increasingly assessing the potential utility of complementing recruitment strategies with social media and may benefit from our real-world data demonstrating the public’s interest in trial opportunities. Bottom line costs for recruiting through digital strategies can be difficult to tease out in individual studies because of the impact of study inclusion criteria, geographical region, staff time needed, etc. However, of those studies that compared the costs of recruiting via Facebook, for example, as compared with traditional media, Facebook was usually less expensive or at least comparable. This was especially true for recruiting hard-to-reach or rare disease populations [51–55]. Thus, allocation of funding to digital awareness strategies may be a worthwhile investment by studies seeking to capitalize on the increasing reach of these kinds of media in service of improved study recruitment.

### Lessons Learned

Digital media messaging was extremely cost-effective for promoting our trials website, and video awareness messages were particularly effective in terms of reach. Integrating this online messaging with other media channels further magnified the effects of our efforts. For example, including a promotion of our online tool in a newsletter emailed to our in-house mailing list during the media campaign spiked site visits dramatically. In addition, designing our messages in a format consistent with PSAs, then posting these to a PSA clearinghouse, allowed our messages to be picked up by national print media and promoted at no additional cost to us, resulting in an exponential increase in our reach.

We also learned that the effect of our campaign was not durable, i.e., few online visitors returned to the website. When recruiting for a clinical trial, this may not be a drawback—potential participants who navigate to the trial site may need only a single visit to determine if they are eligible and interested or not. If return visits to the site are desirable, the use of multiple impressions via various media channels, with social media as the centerpiece, may be productive. These issues, including strategies and factors that may facilitate ongoing engagement, are worth further exploration in various research contexts.

### Limitations

While the number of individual study records viewed by Trials Today users is encouraging, our exploration focused on generating increased usage of Trials Today and did not assess how many site visitors actually went on to pursue participation in a clinical trial they found using Trials Today. We are currently pursuing study-specific awareness implementations that will be able to connect our promotional efforts to key recruitment metrics such as numbers of participants contacting the study personnel and the number deciding to participate. This approach will allow us to evaluate the utility of a medium like Facebook for increasing participation in clinical trials, one of the guiding goals of the Trials Today tool.

Another issue that we observed is that increased usage did not persist after the end of the campaign. However, the fact that usage was not repeated may simply indicate that users found the trials they sought. Our ultimate goal is to increase clinical trial participation. For some users, this may require following the site regularly to view new trials as they are added, while for many others, a single use may be sufficient. In any event, it is unlikely that all users found what they needed during the promotional period, and therefore we may need repeated, long-term exposure to our messaging to serve those individuals whose need for a trial is more difficult to satisfy. Keeping the tool in the public eye will likely require ongoing promotion to catch people at the time they have an active need for the tool. Future work will explore opportunities to engage visitors in ways that foster ongoing awareness of the site. In addition, further study-specific investigations that include individuals from trial inquiry through study enrollment and completion will allow us to further assess the utility of the tool for individual participants and studies.

### Recent Improvements and Future Directions

Since the Trials Today promotion took place, we have instituted additional improvements to the tool. We now show Trials Today studies in RM participant-facing dashboards for easier access. We have also helped close the loop in communication between researchers and participants by listing a study team contact name and email address or phone number for each trial. Our team is currently exploring approaches for further facilitating contact between interested individuals and study investigators by offering an additional feature to allow interested individuals to send their contact information to research contacts for Trials Today studies in which they potentially want to participate.

We plan to evaluate outcomes from targeted Facebook messaging in future research. One pilot study we currently have in process uses Facebook messaging to recruit participants to a clinical trial with narrow inclusion criteria that will investigate the effects of a medication for rheumatoid arthritis. Success will be measured by a comparison of enrollment rates pre- and post-messaging. Future work will also explore utility of multi-channel campaigns (e.g., those distributing awareness strategies through both Facebook and Instagram) and evaluation of different types of campaign metrics, including Facebook data such as impressions, reach, and user engagement characteristics (comments, shares, likes) as well as data more proximal to trial recruitment, such as the volume of potential participants contacting a study team and the number recruited to the study.

We are also planning a usability study to determine other ways in which the Trials Today tool could be made more accessible and user-friendly. There may be a need, for example, to assist trial-seekers more extensively with medical terminology and with understanding inclusion/exclusion criteria.

## Conclusions

Many people are eager to learn of clinical trials that are researching new treatments for chronic diseases, addictions, or mental illnesses. Successful recruitment and completion of such studies depends on strong connections between potential participants and study staff. Awareness of research opportunities among the general public is a critical first step, and online digital media is a cost-effective means. Integrating video and other media channels, such as print media and email list outreach, into a social media campaign can magnify efforts and reduce the cost-per-participant further. Couching the messaging in a PSA format can also yield substantial benefits.

## Supplementary material

For supplementary material accompanying this paper visit https://doi.org/10.1017/cts.2019.404. click here to view supplementary material
