# Supplementary material for: To end disease tomorrow, begin with trials today: Digital strategies for increased awareness of a clinical trials finder
Source: J Clin Transl Sci. 2019 Aug 20;3(4):190–8. doi: 10.1017/cts.2019.404 (PMC6799228; doi:10.1017/cts.2019.404)
Supplement: Supplementary file 1 [file S2059866119004047sup001.docx]

**Supplemental Information**

**Supplemental Table 1. Banner Performance Metrics for Topics by City--Impressions/Clicks (CTR-Click through Rate)**

|  | **Phase I**  **5/22/17 – 7/30/17** | | | | | **Phase II**  **8/1/17 – 9/30/17** | | | | **TOTALS** |
| --- | --- | --- | --- | --- | --- | --- | --- | --- | --- | --- |
| **TOPIC** | **San Antonio, TX** | **Buffalo, NY** | **Portland, OR** | **Cincinnati, OH** | **Wash, DC** | **Sacra-mento, CA** | **Birming-ham, AL** | **Miami, FL** | **St. Louis, MO** |  |
| **Diabetes** | 501,678/1,455  (0.29%) | 753,020/2,167  (0.29%) | 650,953/1,737  (0.27%) | 508,879/1,463  (0.29%) | 756,786/2,127  (0.28%) | 319,681/904 (0.28%) | 320,571/868 (0.27%) | 293,230/875 (0.30%) | 293,097/525 (0.18%) | 4,397,895/12,121  (0.27%) |
| **Heart Disease** | 505,602/1,399  (0.28%) | -- | 654,335/1,787  (0.27%) | 507,096/1,447  (0.29%) | 757,511/2,149  (0.28%) | 325,019/902 (0.28%) | 318,990/879 (0.28%) | 294,802/804 (0.27%) | 293,741/416 (0.14%) | 3,657,096/9,783  (0.26%) |
| **Kidney Disease** | 501,483/1,405  (0.28%) | 650,912/1,719  (0.26%) | 651,294/1,715  (0.26%) | 510,019/1,433  (0.28%) | 752,545/2,034  (0.27%) | -- | -- | -- | -- | 3,066,253/8,306  (0.27%) |
| **Crohn’s Disease** | 300,553/ 827  (0.28%) | 653,244/1,821  (0.28%) | 350,679/1,032  (0.29%) | 305,844/877  (0.29%) | -- | -- | -- | -- | -- | 1,610,320/4,557  (0.29%) |
| **Alzheimer’s Disease** | 300,569/ 829  (0.28%) | 600,842/1,668  (0.28%) | 450,755/1,521  (0.34%) | 302,389/792  (0.26%) | -- | 316,484/872 (0.28%) | -- | 299,576/879 (0.29%) | 293,018/544 (0.19%) | 2,563,633/7,105  (0.27%) |
| **Asthma** | 501,340/1,448  (0.29%) | -- | -- | 509,207/1,477  (0.29%) | -- | -- | -- | -- | -- | 1,010,547/2,925  (0.29%) |
| **COPD** | 351,224/1,167  (0.33%) | -- | -- | 358,416/1,041  (0.29%) | -- | -- | -- | -- | -- | 709,640/2,208  (0.31%) |
| **Pulmonary Sarcoidosis** | -- | -- | -- | 228,062/623  (0.27%) | -- | -- | -- | -- | -- | 228,062/623  (0.27%) |
| **Lupus** | -- | -- | -- | -- | -- | -- | 323,384/905 (0.28%) | 294,201/852 (0.29%) | 293,063/637 (0.22%) | 910,648/2,394  (0.26%) |
| **General Concepts** | -- | -- | -- | -- | -- | 248,059/689 (0.28%) | 257,337/676  (0.28%) | 245,808/681 (0.28%) | 248,044/394 (0.16%) | 999,248/2,440  (0.25%) |

**Supplemental Table 2. Facebook Performance Metrics for Topics by Market—Impressions/Clicks (CTR-Click through Rate)**

|  | **Phase I**  **5/22/17 – 7/30/17** | | | | | **Phase II**  **8/1/17 – 9/30/17** | | | | **TOTALS** |
| --- | --- | --- | --- | --- | --- | --- | --- | --- | --- | --- |
| **TOPIC** | **San Antonio, TX** | **Buffalo, NY** | **Portland, OR** | **Cincinnati, OH** | **Wash, DC** | **Sacra-mento, CA** | **Birming-ham, AL** | **Miami, FL** | **St. Louis, OH** |  |
| **Diabetes** | 684,628/20,441  (2.99%) | 204,604/5,384  (2.63%) | 204,746/4,013  (1.96%) | 252,007/5,523  (2.19%) | 71,204/2,274  (3.19%) | 124,174/1,927  (1.55%) | 129,328/2,255  (1.74%) | 248,369/5,394  (2.17%) | 138,165/2,234  (1.62%) | 2,057,225/49,445  (2.23%) |
| **Heart Disease** | 431,645/8,727  (2.02%) | -- | 248,689/3,507  (1.41%) | 333,484/5,396  (1.62%) | 93,802/2,732  (2.91%) | 158,103/1,977  (1.25%) | 152,232/2,709  (1.78%) | 316,880/6,510  (2.05%) | 194,436/2,795  (1.44%) | 1,929,271/34,353  (1.81%) |
| **Kidney Disease** | 183,812/3,901  (2.12%) | 105,571/1,942  (1.84%) | 136,984/1,845  (1.35%) | 143,571/2,194  (1.53%) | 41,278/1,024  (2.48%) | -- | -- | -- | -- | 611,216/10,906  (1.86%) |
| **Crohn’s Disease** | 54,810/753  (1.37%) | 50,794/518  (1.02%) | 77,565/677  (0.87%) | 90,688/936  (1.03%) | -- | -- | -- | -- | -- | 273,857/2,884  (1.07%) |
| **Alzheimer’s** | 124,111/2,930  (2.36%) | 54,630/1,332  (2.44%) | 104,063/1,574  (1.51%) | 98,978/1,455  (1.47%) | -- | 142,213/1,815  (1.28%) | -- | 139,091/2,171  (1.56%) | 193,571/2,963  (1.53%) | 856,657/14,240  (1.74%) |
| **Asthma** | 109,234/2,913  (2.67%) | -- | -- | 82,197/1,174  (1.43%) | -- | -- | -- | -- | -- | 191,431/4,087  (2.05%) |
| **COPD** | 66,412/2,121  (3.19%) | -- | -- | 53,158/1,246  (2.34%) | -- | -- | -- | -- | -- | 119,570/3,367  (2.77%) |
| **Lupus** | -- | -- | -- | -- | -- | -- | 62,402/434  (0.70%) | 127,795/845  (0.66%) | 139,764/1,098  (0.79%) | 329,961/2,377  (0.72%) |
| **General Concepts** | -- | -- | -- | -- | -- | 202,305/2,915  (1.44%) | 222,298/4,118  (1.85%) | 270,796/7,473  (2.76%) | 242,712/3,917  (1.61%) | 938,111/18,423  (1.92%) |

**Supplemental Figures: Trials Today User Survey Data**

*Percentages equal more than 100% due to multiple responses. accepted.
